# Recent Advances in Trajectory Planning and Object Recognition for Robot Sensing and Control

**DOI:** 10.3390/s24144509

**Published:** 2024-07-12

**Authors:** Gang Peng, Zhun Fan

**Affiliations:** 1 School of Artificial Intelligence and Automation, Huazhong University of Science and Technology, Wuhan 430074, China; 2 Shenzhen Institute for Advanced Study, University of Electronic Science and Technology of China, Shenzhen 518110, China; fanzhun@uestc.edu.cn

## 1. Trajectory Planning and Control

Nowadays, trajectory planning and control technology has been extensively utilized in industry, agriculture, service, construction, infrastructure inspection, military reconnaissance, disaster management, and other areas. However, the complex natural environment can significantly impact the accuracy of trajectory planning, and developing this technology entails overcoming substantial challenges. This Special Issue of the journal *Sensors* presents three articles on trajectory planning and control that are of significant importance for advancing this technology.

To address the issue of intricate environmental and dynamic limitations in real-world applications, the article [contribution 1] proposes a novel gradient potential field, termed the kino-gene regulatory network (K-GRN) potential field, which is based on a GRN model. By enhancing the gene regulation network model, the potential field becomes adaptable to the UAV’s dynamic conditions and its surroundings, thereby extending the GRN into a kinodynamic GRN (K-GRN). The amalgamation of environmental insights and kinodynamic constraints within the potential field framework bolsters the adaptability and stability of the generated trajectories.

To improve the comprehensiveness of path planning, the article [contribution 2] proposes a framework for autonomous UAV-based landmine detection to determine the coverage route for scanning the target area. This method extracts the area of interest using segmentation based on deep learning and then constructs the coverage route plan for the aerial survey. Multiple coverage path patterns are used to identify the ideal UAV route. The effectiveness of the suggested framework is evaluated using several target areas of differing sizes and complexities.

In article [contribution 3], researchers design a stepped rotor BLSRM structure and combined particle swarm optimization (PSO) algorithm with finite element analysis to optimize the structural parameters of the motor. Subsequently, a performance analysis of the original and new motors was conducted using finite element analysis software, and the results showed that the stepped rotor BLSRM had an improved self-starting ability and significantly reduced torque fluctuation, verifying the effectiveness of the proposed motor structure and optimization method. 

These three papers illustrate the continual development and innovation in trajectory planning and control technology.

## 2. Environment Perception and Target Detection

Environment perception and target detection, using deep learning, image processing, and other technologies, have a wide range of applications in medicine, sports, and other fields. In recent years, environmental perception and target detection technology has gained increasing attention and research. In this article, we introduce four new methods to enhance the accuracy of environment perception and target detection technology from different perspectives.

The article [contribution 4] proposes a novel human motion prediction method that utilizes dual-attention and multi-granularity temporal convolutional networks. By designing a dual-attention model for extracting inter-joint and intra-joint spatial features, provide richer information sources for motion prediction. Then, a multi-granularity temporal convolutional network is introduced to realize the discriminant fusion of different time granularities, flexibly capturing complex short-term and long-term temporal dependencies, and thereby further improving the model’s performance. Article [contribution 5] proposes an evaluation model based on contrastive learning to evaluate the neurological function of patients with Alzheimer’s disease. It introduces an objective dementia severity scale based on MRI using a contrastive learning framework to evaluate neurological function during Alzheimer’s disease progression and can accurately discriminate between different stages of Alzheimer’s disease progression. The article [contribution 6] focuses on an improved version of the H-GrabCut image segmentation algorithm to address the aforementioned issues and achieve background removal in complex pedestrian backgrounds. By incorporating the Bilateral-MSRCR image enhancement algorithm and introducing two-dimensional information entropy, UV component distance, and LBP texture features, it effectively resolves the resource limitations associated with nesting multiple deep learning algorithms while enabling more accurate distance measurement between robots and pedestrians. Furthermore, the article [contribution 7] designs an infrared small target detection method based on an attention mechanism. It proposes a top–down active attention module to obtain target knowledge–experience Gaussian shape features to improve the signal-to-clutter ratio gain and background suppression factor and the decision feedback equalization in order to find real targets to adapt to environmental changes in different scenarios. The proposed method can achieve better detection performance than conventional baseline methods and mathematical proofs are provided to validate the proposed method.

We believe that these research results will provide robust support for applying and promoting environment perception and target detection technology.

## 3. Multi-Sensor Fusion and Calibration

Multi-sensor fusion and calibration are critical for enhancing the accuracy and robustness of various robotic and sensing systems. This integration allows for the seamless combination of data from different sensors, enabling more reliable perception and detection capabilities. This article introduces three innovative methods aimed at improving the accuracy and robustness of multi-sensor fusion and calibration techniques.

In the realm of multi-sensor fusion and calibration, three notable contributions have been made. The paper [contribution 8] introduces the VILO algorithm, which addresses challenges faced by existing visual–inertial SLAM algorithms by tightly coupling vision, IMU, and 2D LiDAR observations, resulting in improved accuracy and robustness, particularly in environments with insufficient visual features. The paper [contribution 9] presents a novel tool frame calibration method tailored for ultrasonic testing systems, utilizing reflectors to enhance precision without additional measuring devices, thus overcoming limitations in underwater environments. Lastly, the paper [contribution 10] introduces a new method for hand–eye calibration error analysis and algorithm optimization, leveraging augmented reality markers to achieve higher precision calibration results. These advancements significantly enhance the accuracy and robustness of robotic systems across diverse applications, contributing to the ongoing development of environment perception and target detection technologies.

In conclusion, the advancements in multi-sensor fusion and calibration techniques, as demonstrated in articles [contributions 8–10], significantly improve the accuracy and robustness of robotic systems across diverse applications. These methodologies contribute to the ongoing development of environment perception and target detection technologies, facilitating their integration into real-world scenarios.

## 4. Autonomous Unmanned System

Autonomous unmanned systems represent a crucial frontier in modern technology, offering innovative solutions across various domains. These systems are instrumental in addressing complex challenges and enhancing efficiency in critical tasks, ranging from underwater exploration to disaster response and beyond.

The article [contribution 11] addresses challenges in underwater imaging, proposing a method based on the dark channel prior (DCP) to enhance visibility and eliminate color cast effectively. [Contribution 12] presents a nonlinear model predictive control (NMPC) scheme for underwater biomimetic vehicle-manipulator systems (UBVMS), enabling robust path following and dynamic obstacle avoidance. The article [contribution 13] proposes an autonomous optimal deployment method for mobile base stations in high-rise building fire environments, enhancing rescue operations through accurate positioning and collision avoidance. Furthermore, the article [contribution 14] introduces a novel recovery system for unmanned surface vehicles (USVs), incorporating physical modeling of disturbance forces and optimized genetic algorithms to enhance recovery efficiency and accuracy.

In conclusion, the advancements presented in refs. [contributions 11–14] contribute significantly to the development and effectiveness of autonomous unmanned systems. These methodologies address critical challenges and offer innovative solutions, enhancing capabilities and improving performance in real-world applications.

## 5. Conclusions

The theme of this Special Issue is trajectory planning and target recognition for robot sensing and control. This Special Issue selected 14 articles, which can be divided into the following four categories: trajectory planning and control [contribution 1–3], environmental perception and target detection [contributions 4–7], multi-sensor fusion and calibration [contributions 8–10], and autonomous unmanned systems [contributions 11–14]. The research objects cover UAV, human posture, underwater bionic aircraft, multi-sensor fusion, etc. The development of artificial intelligence algorithms provides a new solution for trajectory planning and target recognition of robot sensing and control and shows great potential in dealing with complex environments and dynamic constraints.

